# Advances in Biological Function and Clinical Application of Small Extracellular Vesicle Membrane Proteins

**DOI:** 10.3389/fonc.2021.675940

**Published:** 2021-05-20

**Authors:** Defa Huang, Jie Chen, Die Hu, Fangfang Xie, Tong Yang, Zhengzhe Li, Xiaoxing Wang, Yongwei Xiao, Jianing Zhong, Yu Jiang, Xiaokang Zhang, Tianyu Zhong

**Affiliations:** ^1^ The First School of Clinical Medicine, Gannan Medical University, Ganzhou, China; ^2^ Laboratory Medicine, First Affiliated Hospital of Gannan Medical University, Ganzhou, China; ^3^ Key Laboratory of Prevention and Treatment of Cardiovascular and Cerebrovascular Diseases, Ministry of Education, Gannan Medical University, Ganzhou, China; ^4^ Department of Pharmacology and Chemical Biology, University of Pittsburgh School of Medicine, Pittsburgh, PA, United States; ^5^ Department of Preventive Medicine, Gannan Medical University, Ganzhou, China; ^6^ Precision Medicine Center, First Affiliated Hospital of Gannan Medical University, Ganzhou, China

**Keywords:** small extracellular vesicles (sEVs), exosomes, membrane protein, biomarker, diagnosis, targeted therapy

## Abstract

Small extracellular vesicles are membrane-bound vesicles secreted into extracellular spaces by virtually all types of cells. These carry a large number of membrane proteins on their surface that are incorporated during their biogenesis in cells. The composition of the membrane proteins hence bears the signature of the cells from which they originate. Recent studies have suggested that the proteins on these small extracellular vesicles can serve as biomarkers and target proteins for the diagnosis and treatment of diseases. This article classifies small extracellular vesicle membrane proteins and summarizes their pathophysiological functions in the diagnosis and treatment of diseases.

## Introduction

Small extracellular vesicles (sEVs) are membrane-bound vesicular structures produced by the endosomal pathway (exosomes) and others independent of the endosomal pathway (1). Traditionally, exosomes are defined as a subset of sEVs with a diameter of 30-200 nm and a density of 1.12-1.18 g/ml (2). These vesicles are found in a wide variety of body fluids, such as blood, urine, breast milk, ascites, amniotic fluid, saliva and cerebrospinal fluid (3–5). They can be isolated and separated from other membrane-bound EVs through differential centrifugation, precipitation and immunoassays (6). These approaches are based on the differences in size, density, and surface markers of EVs. However, the vesicles obtained by the above approaches have a great heterogeneity, hence difficult to determine their pathways of origin and composition of surface proteins. Therefore, International Society for Extracellular Vesicles (ISEV) published the Minimal information for studies of extracellular vesicles 2018 (MISEV2018) (7) that recommend the use of “small EVs (sEVs)” as the current term. Meanwhile, we discriminate the different extracellular vesicles based on vesicle membrane proteins (1, 8–10) (**Table 1**).

**Table 1:** Classification of different extracellular vesicles based on vesicle membrane proteins.

Name	Diameter	Surface marker	Source	References
Subpopulations of EVs				
Large EVs	Majority larger than 150 nm	Actinin-4 and mitofilin	human primary monocyte-derived dendritic cells	(1)
Medium-sized EVs	A mean size above 200 nm			
Small EVs	A mean size below 200 nm	Syntenin-1, EHD4, ADAM10, and Annexin XI		
Subpopulations of sEVs				
CTB-EVs	50-100 nm	Cholera toxin B chain	Mesenchymal stem cell	(8)
AV-EVs		Annexin V		
ST-EVs		Shiga Toxin		
A33-Exosomes	40-60 nm	A33	LIM1863 colon carcinoma cell	(9)
EpCAM-Exosomes		EpCAM		
Large exosome vesicles	90-120 nm	Annexins, ESCRT components, Hsp40 family proteins, signaling transducer G protein subunits, integrins, Rab proteins	Melanoma cells and tissues	(10)
Small exosome vesicles	60-80 nm			
Exomeres	<50 nm (~35 nm)	Non-membranous nanoparticles		

sEVs are rich in various biologically active molecules such as RNA, DNA, and proteins, which can be delivered to target cells through sEVs and affect their functions. Most previous studies have focused on the contents of sEVs (such as nucleic acids and proteins), which are considered to be responsible for the regulatory effects of sEVs (11–13). For example, sEVs containing neurotransmitter receptor proteins released by cortical neuronal cells can enhance the activity of glutamatergic neurons (14). miRNAs in breast cancer cell-released sEVs are found to promote cancer cell metastasis and angiogenesis (15). miRNAs in sEVs secreted by tumor-associated macrophages are shown to stimulate invasion and metastasis of breast cancer cells (16). In addition to miRNA and other soluble contents of inside sEVs, an increasing body of evidence suggests that the surface membrane proteins of sEVs also play an important role in modulating the functions of targeted cells (17).

sEV outer membrane is the phospholipid bilayer that, contains many different membrane proteins. At present, some specific membrane proteins on the surface of sEV have been used as markers for isolation and identification of sEV. These proteins have also been found to be involved in sEV formation, intercellular interaction, membrane transport, and cell metabolism (18). In recent years, sEV membrane proteins are shown to have a potential in the diagnosis and treatment of diseases. Studies have shown that CD82 from sEVs derived from serum or plasma can be used as a diagnostic biomarker for breast cancer (19). The membrane protein CD9 from mouse hepatocellular sEVs can be fused with the therapeutic miRNA, allowing the miRNA to be encapsulated in sEVs and delivered to hepatocellular carcinoma cells for treatment (20). Although some studies have reported the role of sEV membrane proteins (21), their classification is not clear. Understanding the classification and biological functions of sEV surface membrane proteins are of great significance for further study of sEV.

## sEV Membrane Protein Classification

**Figure 1:**
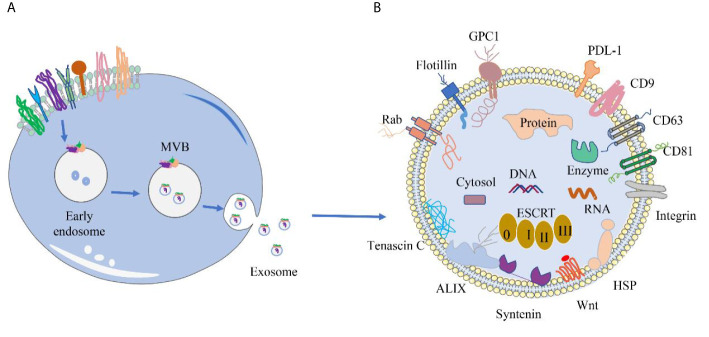
Origin of sEVs and distribution of different sEV membrane proteins. **(A)** Origin of sEVs. sEVs are produced through the exosomal pathway. First, plasma membrane invagination of donor cells forms early endosomes, which further mature into late endosomes. During the maturation, the membrane of early endosomes invaginates inwardly to form ILVs. Endosomes with ILVs are often referred to as MVBs. When MVBs fuse with the plasma membrane, ILVs are released into the extracellular space and termed as sEVs. **(B)** Common membrane proteins of sEVs. Tetraspanin proteins (CD9, CD63, CD81), PD-L1, Integrins; Wnt protein, ALIX, Syntenin, HSPs, tenascin C; GPC1, Rabs, Flotillin, etc.

The membrane proteins of sEVs are originated from the endocytic membrane compartments that give rise to the sEVs (22) (**Figure 1A**). Inward invagination of early endosomal membrane results in formation of intraluminal vesicles (ILV) inside the endosomal compartments, which are termed as multivesicular bodies (MVBs). During the invagination, proteins and RNAs are selectively packed into ILVs through endosomal sorting complex required for transport (ESCRT)-dependent pathway or ESCRT-independent pathway. When MVBs fuse with the plasma membrane, ILVs are released into the extracellular space as sEVs. The membrane proteins of sEV include integral membrane proteins, peripheral membrane proteins and lipid anchoring membrane proteins (**Figure 1B**). Many of the membrane proteins are components of ESCRT complexes that are incorporated into sEVs during the formation of ILVs. Others are originated from the plasma membranes and involved in cell-to-cell communication, and hence responsible for producing cellular effects in the targeted cells. **Table 2** describes the expression location and biological effects of sEV membrane proteins.

**Table 2:** Expression location and biological function of sEV membrane proteins.

Name	Source	Biological role	References
CD9	Ovarian cancer cells, T cells, dendritic cells, hepatocyte	Regulate tumor progression, regulate antigen presentation	(23–26)
CD82	Renal cancer cells, prostate cancer cells	Inhibit tumor development	(27)
CD151	Breast cancer cells, pancreatic cancer cells	Promote tumor progression	(28, 29)
CD81	T cells	Activate T cells, strengthen antigen composition	(30)
CD37	Dendritic cells	Enhanced antigen presentation	(31)
PD-L1	Glioblastoma cells, non-small cell lung cancer, oral cell carcinoma, melanoma cells, prostate cancer cells, substantive organs head and neck cancer cells	Mediate tumor immune escape and promote tumor development, mediates resistance to immunotherapy, reduction of allograft immune response	(32–33)
Integrin	Ovarian tumor cells, breast cancer cells	Promote tumor metastasis, prediction of tumor metastasis	(34–35)
Wnt	Breast cancer cells, fibroblasts, cardiac progenitor cell, endothelial cells	Enhance tumor metastasis, maintain cardiac function, impact sEVs release	(36–38–39)
ALIX	All sEVs	Participate in the formation of sEVs	(40)
Syntenin	All sEVs	Participate in the sorting of sEVs content	(41, 42)
HSPs	Cardiomyocytes, oral squamous cell carcinoma, colorectal cancer cell	Increased blood supply to the heart, regulate tumor progression	(43–44)
Bone morphogenetic protein, TGFR-β1, Tenascin C	Bone marrow stem cells, osteoblasts and osteoclasts	Promote bone formation and differentiation	(45, 46)
Glypican-1	Colorectal cancer, pancreatic cancer, breast cancer cell	Enhance tumor cell invasion and metastasis	(47–48)
Rab27a Rab27b Rab11	Chronic myeloid leukemia cells, breast cancer cells	Regulate sEVs release, promote tumor metastasis	(49–50)
Flotillin	Prostate cancer cells	Change the composition of sEVs	(51, 52)

### Integral Membrane Proteins

Integral membrane proteins, also known as intrinsic membrane proteins, are a type of proteins that are partially or completely embedded in a phospholipid bilayer through a single or multiple membrane spans. sEV integral membrane proteins mainly include tetraspanin proteins, programmed death ligand 1 and integrins.

#### Tetraspanin Proteins

Tetraspanin proteins are common integral membrane proteins with four transmembrane spans (53). Some tetraspanin proteins were highly enriched in sEV membrane, including CD9, CD63, CD37, CD53, CD81, CD82, CD151, and so on. Among them, CD9, CD63 and CD81 are stably expressed in sEVs derived from all tissue cells, and are commonly used as markers for the isolation and identification of sEVs (54).

Recent studies have shown that tetraspanin proteins in some sEVs are involved tumor metastasis. CD9 of sEVs derived from the serum of patients with ovarian cancer is able to interact with a variety of proteins including integrins and immunoglobulin superfamily (23). Consequently, it promotes adhesion of ovarian cancer cells to surrounding stroma and inhibits their metastasis and, thereby preventing the development of ovarian cancer. The sEVs released by hepatocytes also express CD9. However, in hepatocytes undergoes canceration the levels of CD9 in sEVs is significantly reduced. When CD9 is overexpressed, it inhibits the proliferation of hepatoma cells *in vitro* and *in vivo* (24). CD82 of serum or plasma-derived sEVs from renal cell carcinoma patients has been found to inhibit metastasis of the cancer cells by blocking the TGF-β1/Smad signaling pathway (27). Similarly, CD82 of sEVs in the serum of prostate cancer patients was shown to promote the expression of TBX2 (a tumor suppressor protein) and P21 (a cell cycle inhibitory enzyme), thereby reducing the primary tumor formation foci and ultimately inhibiting the proliferation and migration of prostate cancer cells (55). In contrast, the tetraspanin protein CD151 on some sEVs has a role in promoting tumor growth. It was found that CD151 on serum or plasma-derived sEVs from breast cancer patients promoted breast cancer cell neovascularization and cancer cell metastasis. The pro-cancer effects of CD151 was mediated through interactions with integrins (α3β1, α6β1) growth factor receptors, and matrix metalloproteinase (MMP-2) (28). Similarly, CD151 of sEVs secreted by pancreatic cancer cells was found to promote the metastasis of pancreatic cancer in rats through activating stromal cells and increasing the expression of inflammatory cytokines in hematopoietic cells (29).

Additionally, some sEV tetraspanin proteins play an important role in antigen presentation. CD81 on T cell-derived sEVs was found to accelerate the maturation of immune synapses on T cells and ensure T-cell activation by interacting with intercellular adhesion molecules (30). Likewise, CD9 of sEVs released by T cells was shown to promote the formation of immune synapses by regulating the function of integrin β1 on T cells, thereby enhancing antigen presentation (25). Notably, the tetraspanin proteins of some sEVs can interact with MHC molecules to enhance antigen presentation. A study showed that CD9 from sEVs secreted by dendritic cells (DCs), by forming specialized clusters with MHC-II, allowed DCs to stimulate naive T-cell activation more efficiently, thereby enhancing antigen presentation (26). Similarly, CD37 of DCs-derived sEVs was reported to play a role in MHC aggregation and is involved in peptide/MHC presentation. This may be achieved by regulating the interaction of the MHC with other tetrameric proteins that promote MHC aggregation, such as CD9 and CD82 (31).

#### Programmed Death Ligand 1

Programmed death ligand 1 (PD-L1), a transmembrane protein highly expressed on the surface of tumor cells, inhibits T-cell function by binding to programmed death receptor 1 (PD-1), leading to tumor immune escape (56, 57). Moon et al. (58) demonstrated the presence of PD-L1 on sEVs of human urine or plasma origin. A study showed that glioblastoma-released sEVs expressing PD-L1 inhibited the activation and proliferation of CD4^+^ T and CD8^+^ T cells by binding to PD-1, leading to immune escape of glioblastoma cells (32). Another study found that PD-L1 on sEVs of non-small cell lung cancer origin promoted the growth of these tumor cells by binding to PD-1 to reduce T-cell activity, thereby inhibiting T-cell secretion of interferon-γ (59). It was also found that sEVs isolated from the serum of oral cancer mice expressing PD-L1 prevented the infiltration of CD4^+^ T and CD8^+^ T cells into the tumor microenvironment by binding PD-1, hence enhancing the proliferation and metastasis of these tumor cells (60).

Furthermore, PD-L1 on sEVs mediates resistance to immunotherapy by direct binding of anti-PD-L1 antibodies. Chen et al. (61) discovered that PD-L1 on metastatic melanoma-derived sEVs inhibited CD8^+^ T cell activation and promoted tumor growth, but treatment with anti-PD-1 antibodies blocked this effect. In a mouse model of prostate cancer, treatment with anti-PD-L1 antibodies was ineffective because of the presence of PD-L1 on sEVs (62). Likewise, in patients with head and neck cancer, sEVs in the serum of PD-L1 positive patients significantly inhibited CD69 on CD8^+^ T cells, which was blocked by anti-PD-1 antibodies (63). These results indicate that the PD-L1 of sEV plays an important role in anti-PD-L1/PD-1 therapy.

Interestingly, in allografts sEV PD-L1/PD-1 plays an important role in the regulation of the alloimmune response. PD-L1 binds to PD-1 to inhibit T-cell activation in experimental models of skin and heart transplantation and in experimental models of graft-versus-host disease, thereby reducing the incidence of graft rejection (64–66). In a cardiac allograft model, treatment with PD-L1-Ig was associated with prolonged allograft survival and reduced lymphocytic infiltrate in the graft (33).

#### Integrins

Integrins are transmembrane heterodimeric glycoproteins composed of α and β subunits. The integrins of sEVs derived from some tumor cells may contribute to the formation of pre-metastasis niches and thus promote tumor metastasis. Feng et al. (34) discovered that integrins (α5β1, αvβ3) on the membranes of ovarian tumor cell-derived sEVs could modulate the intracellular matrix and mediate communication between ovarian tumor cells, tumor-associated fibroblasts, and local immune cells. This would further enhance the formation of the tumor microenvironment, thereby promoting the formation of the pre-metastasis niche of ovarian cancer. Another study showed that integrin α3β1 on sEVs secreted by breast cancer cells directly interacted with CD151 in tumor stromal cells, thereby mediating their metastasis to distant sites (67). When α3β1 was knocked down in breast cancer cells, the ability of the cancer cells for metastasis was significantly inhibited. In addition, some integrins on the membrane of sEVs may be used for predicting tumor cell organ-specific metastasis. Hoshino et al. (35) found that the levels of the integrins ITGβ4 and ITGαv in the serum sEVs of patients with breast and pancreatic cancer were significantly increased after the development of lung and liver metastases. This result suggests that specific sEVs integrins in the serum of patients with breast and pancreatic cancer may predict the site of tumor metastasis.

### Peripheral Membrane Proteins

Peripheral membrane proteins are a class of proteins that rely on ionic bonding to the hydrophilic portion of proteins or lipid molecules on the membrane surface. Wnt proteins, scaffold proteins, heat shock proteins and bone morphogenetic-related proteins are common peripheral membrane proteins of sEV.

#### Wnt Proteins

Wnt proteins refer to a class of secreted glycoproteins that are involved in the Wnt/β-catenin pathway or the Wnt/planar cell polarity (PCP) pathway. Wnt proteins are closely related to the biogenesis of sEVs, tumor invasion and metastasis. Hesam et al. (36) showed that human endothelial cells stimulated the expression of CD63, Alix, Rab27a and Rab27b genes through autophagy and Wnt signaling, which increased the release of sEVs. Chen et al. (37) demonstrated that Wnt10b of sEVs released by breast cancer cells induced epithelial-mesenchymal transformation (EMT) by activating the Wnt/β-catenin signaling pathway, thereby promoting metastasis of breast cancer cells. EMT is an important process in the metastasis of tumor cells, which transforms epithelial cells into mesenchymal cells, thereby reducing cell polarity and adhesion and gaining migration ability (68). Furthermore, Wnt proteins from sEVs released by tumor-associated fibroblasts also promote invasion and metastasis of breast cancer cells through activation of the Wnt-PCP signaling pathway (38). Hu et al. (69) found that the Wnt protein of fibroblast-derived sEVs activated the Wnt/β-catenin signaling pathway in colorectal cancer (CRC) stem cells, leading to drug resistance in CRC cells. Interestingly, cardiac progenitor cell-derived sEVs carry Wnt proteins that promote cardiac angiogenesis and maintain homeostasis of cardiac function by activating Wnt/β-catenin signaling (39).

#### Scaffold Proteins

The scaffold proteins on sEV are a type of proteins enriched in the phospholipid inner membrane layer, which exert function by interacting with other proteins or lipids. ALIX, a common scaffolding protein on sEV, is both a marker protein for sEV and an auxiliary protein of the ESCRT. The ESCRT complexes consist of ESCRT-0, ESCRT-I, ESCRT-II, and ESCRT-III (70). ALIX interacted with the charged multivesicular body protein 4 in ESCRT-III to drive vesicle shedding (40). Syntenin is another scaffolding protein on the peripheral membrane proteins of sEV. Overexpression of Syntenin on the sEV membrane reduced the constitutive endocytosis rate of CD63 (71). Additionally, Syntenin was able to interact with Syndecans (a proteoglycan) and further connected with ALIX to participate in the sorting of RNA and protein into these particles (41, 42).

#### Heat Shock Proteins

Recently, some heat shock proteins (HSPs) have also been found to be important peripheral proteins of sEV. HSPs are recognized as an intrinsic protective agent of cardiomyocytes against stress. It has been reported that HSP70 on sEVs derived from rat and human plasma prevented cardiac ischemia and reperfusion injury by activating toll-like receptor 4 and MAPK signaling pathways in myocardial cells of rats with myocardial infarction (43). Zhang et al. (72) showed that HSP20 of sEVs secreted by mouse cardiomyocytes promoted myocardial angiogenesis and enhanced cardiac blood supply by activating the growth factor receptor signaling cascade response in these cells.

In addition, the peripheral membrane HSPs of sEV have also been found to be associated with tumor progression. A study found that HSP90 was highly expressed in sEVs derived from oral squamous cell carcinoma (OSCC), and knocking down HSP90 reduced the survival rate of these cells (73). Another study showed that HSP60 was highly expressed in plasma sEVs in patients with CRC, and found that HSP60 was related to CRC cell proliferation, invasion and migration (44).

#### Bone Morphogenesis Related Proteins

Bone morphogenesis-related proteins have also been shown to exist on the membrane of sEV. These proteins play important functions in the formation and differentiation of bone cells. Studies have shown that in the bone remodeling microenvironment, bone marrow stem cells, osteoblasts, osteoclasts, etc. can release sEVs and deliver sEV peripheral membrane proteins, bone morphogenetic proteins and transforming growth factor receptor β1, to osteoblasts, hence regulating osteogenesis (45). Similarly, it has been reported that the peripheral membrane protein “tenascin C” carried on sEVs of bone marrow stromal cell origin is able to regulate the activity and differentiation of osteoblasts and promotes bone regeneration (46).

### Lipid Anchoring Membrane Proteins

Lipid anchoring membrane proteins are a class of proteins that anchor to the cell membrane through lipid moieties. Among those proteins, glypican-1, prenylated small GTPases, and flotillin have been found to be present on sEV.

#### Glypican-1

Glypican-1 (GPC1), a common lipid-anchored membrane protein on sEV, is overexpressed in a variety of solid tumors and may influence tumor progression. A recent study showed that plasma levels of GPC1^+^ sEVs were elevated in CRC patients (47). Overexpression of GPC1 activated epithelial-mesenchymal transition, which further enhanced CRC cell invasion and migration. Another study showed that GPC1 expression of sEVs released from mouse CRC cells was elevated and that high expression of GPC1 affected the generation of a distant premetastatic niche and metastatic organotropism in CRC (74). Furthermore, Frampton et al. (75) discovered that silencing of GPC1 of pancreatic cancer cell-derived sEVs led to reduced tumor angiogenesis and an attenuation of mitogenic responses, which in turn slowed the growth of these tumor cells. Similarly, breast cancer development was found to be positively correlated with patients’ serum levels of GPC1^+^ sEVs, which stimulated angiogenesis and activate fibroblasts in the cellular matrix (48).

#### Prenylated Small GTPases

Prenylated small GTPases are found to be widely distributed on the membranes of sEV. Rabs are common prenylated small GTPases on sEV, which play an important regulatory role in the release of sEVs. Rab27a and Rab27b proteins were reported to control, docking and fusion of different sEVs to the recipient membranes (49). Savina et al. (76) showed that Rab11 overexpressed on sEVs derived from chronic myeloid leukemia cells inhibited release of sEVs. In addition, Wang et al. (50) found that Rab27a overexpression of sEVs released by breast cancer cells promoted the release of insulin-like growth factor-II. This factor promoted breast cancer cell invasion and metastasis by regulating the functions of vascular endothelial growth factor, tissue proteinase D, matrix metalloproteinase 9, and cyclin D1.

#### Flotillin

Flotillin is a lipid raft-tagged protein that has been shown to be present in the membranes of sEV. It has been reported that the release of sEVs may be regulated by flotillin. One study showed that the combined assembly of flotillin1 and flotillin2 caused changes in cell membrane curvature and the formation of small pore-like invaginations in the plasma membrane, thereby increasing the release of sEVs (51). However, another study found that knockdown of flotillin1 and flotillin2 from prostate cancer cell-derived sEVs did not alter the release of sEVs, but reduced the caveolin-1 and annexin A2 proteins within sEVs. The molecular mechanism of this process remained unclear (52).

## Clinical Applications of sEV Membrane Proteins

At present, a large number of studies have shown that changes in sEV membrane proteins are associated with many disease conditions and can be exploited for diagnosis, prognosis and targeted treatment of diseases.

### Biomarkers

Recent studies suggest that sEV membrane proteins produced by cancer cells are quite different from those produced by normal cells. Some proteins on the sEV membrane may be potential indicators for the diagnosis and prognosis of cancer.

#### Prostate Cancer

Some sEV membrane proteins have been found to be potential markers of prostate cancer. It has been reported that integrin αvβ3 and Trop-2 (an anti-adhesion and migration-promoting transmembrane protein) were co-expressed in plasma sEVs from patients with prostate cancer (77). Among them, the expression level of αvβ3 on the membrane of plasma-derived sEVs was significantly increased. When these particles were co-incubated with αvβ3 negative prostate cancer cells, the level of β3 in receptor cells increased. This suggests that αvβ3 may mediate intercellular communication in the progression of prostate cancer. Recent reports have also shown that sEVs secreted from prostate cancer cells expressing αvβ3 integrin may promote cancer cell migration by forming metastatic niche (35), altering angiogenesis and cell signaling (78). These studies demonstrated that integrin αvβ3 on plasma isolated sEVs may be a biomarker for the diagnosis and prognosis of prostate cancer. Another research indicated that the expression of Trop-2 on the membrane of sEVs produced by invasive prostate cancer cells was up-regulated (79). On these sEVs, Trop-2 binding to α5β1 integrin regulated integrin signal transduction, hence promoting the metastasis of prostate cancer cells with Trop-2 knockdown. Therefore, the combined detection of multiple surface markers such as integrin αvβ3 and Trop-2 may characterize the sEVs produced by prostate cancer cells in the future.

It has been reported that the number of sEVs expressing CD81 and prostate specific antigen (PSA) in plasma of patients with prostate cancer increased significantly (80). The release of these vesicles may be related to the acidic tumor microenvironment. Similarly, Logozzi et al. (81) found that prostate cancer patients could be distinguished from non-prostate cancer patients based on the level of plasma sEVs expression of PSA. Mizutani et al. (82) revealed that prostate-specific membrane antigen (PSMA) was also expressed on the sEV membrane in the plasma of patients with prostate cancer, which might be used to isolate prostate cancer-related sEVs from blood samples. Moreover, Cho et al. (83) found that the expression of epithelial cell adhesion molecule (EpCAM), epidermal growth factor receptor (EGFR), Survivin and insulin-like growth factor 1 receptor (IGF-1R) were significantly increased in sEVs derived from prostate cancer cells. The researchers further established a method for simultaneous multiple *in situ* detection of sEV surface proteins with CD63 quantification in a single reaction, which may be used for the diagnosis of prostate cancer. A recent study discovered that transmembrane protein 256 (TM256) enriched on the sEV membrane in urine of patients with prostate carcinoma was also highly expressed in the tissues of the patients (84). TM256 on sEVs diagnosis the prostate cancer with a sensitivity of 94%. The combination of TM256 and LAMTOR1 (a membrane protein specifically located on the surface of advanced endosomes/lysosomes) on these sEVs can increase the sensitivity to 100%. Further verification revealed that the area under the curve (AUC) of TM256 of sEVs was 0.87 and the AUC of TM256 combined with LAMTOR1 was 0.94 (85).

#### Pancreatic Cancer

Pancreatic cancer is a malignant tumor of digestive tract. Recent evidence has suggested that there is a significant difference in sEV membrane proteins isolated from blood between patients with pancreatic cancer and normal controls, which may be a candidate for the diagnosis of pancreatic cancer. GPC1 overexpressed on serum-derived sEVs has extremely high sensitivity and specificity in prediction of pancreatic cancer. It is capable of distinguishing benign pancreatic disease conditions, early and advanced stages of pancreatic cancer (86). Receiver operating characteristic (ROC) curves showed that the AUC of GPC1^+^ sEVs was 1.0, which was significantly higher than that of carbohydrate antigen 19-9 (CA19-9), a serum marker of pancreatic cancer. At the same time, the expression level of EphA2 on serum sEVs was also significantly increased in patients with pancreatic cancer, and the expression level of these vesicles in advanced patients (III and IV) was significantly higher than that in early patients (I and II) (87). It is worth noting that the ability of EphA2 (AUC=0.94) on serum sEVs to distinguish between pancreatic cancer patients and healthy people was similar to that of CA19-9 (AUC=0.95). But in differentiating prostate cancer from benign pancreatic disease, the AUC of EphA2^+^ sEVs was higher (0.92>0.88). When the two factors were combined, an AUC of 0.97 and 0.94 was achieved, respectively.

In addition, it was found that CD44v6, a transmembrane protein, was highly expressed on sEVs released by pancreatic cancer-initiation cells (CIC), which promoted the migration and invasion of non-CIC (88). In non-CIC, knockdown of CD44v6 downregulated Tspan8, thus inhibiting tumor progression. Interestingly, when these cells were treated with CD44v6^+^ sEVs, they regained their metastatic ability. Another study discovered that pancreatic cancer patients with overexpressed PD-L1 containing serum sEVs (7.8 months) had a significant reduction in postoperative survival compared with patients without these vesicles (17.2 months) (89). These sEV membrane proteins may be potential markers for evaluating the prognosis of patients with pancreatic cancer.

#### Colorectal Cancer

sEV membrane proteins also play an important role in CRC. A recent published study demonstrated that compared with healthy people, patients with CRC have 36 proteins in serum sEVs of up-regulated, while 22 proteins are downregulated (90). Many up-regulated proteins were involved in regulating the metastasis of pre-tumor microenvironment, whereas down-regulated proteins were often associated with tumor growth and cell survival. Huang et al. (91) found that hypoxia stimulated CRC cells to release sEVs, which was rich in membrane protein Wnt4. When the Wnt4 containing vesicles were delivered to normoxic CRC cells, they activated the β-catenin pathway in the cells, which promoted their migration and invasion. Li et al. (92) have suggested that the GPC1 on sEVs in patients’ plasma may be an indicator for the diagnosis and treatment of CRC. The concentration of these GPC1 positive vesicles was more than 10 times higher than that in the plasma of healthy subjects, and the level of GPC1 protein was significantly increased in tumor tissues. However, 2 months after removal of the tumors, the expression of these vesicles and the GPC1 protein on their surface returned to normal. Sun et al. (93) confirmed that Copine-III (CPNE3), a membrane-binding protein, was highly expressed in tissues and plasma of patients with CRC. They suggested that the protein may be a predictor of CRC. The ROC curve showed that the AUC value of CPNE3 was 0.791, which was higher than that of carcinoembryonic antigen (CEA) (0.728). It is worth noting that the AUC value of the combined detection was 0.833. Interestingly, plasma sEVs levels were higher in patients with distant metastasis, and these patients showed lower disease-free and overall survival rates. Furthermore, Merendino et al. (94) found that the expression of Hsp60 on the membrane of sEVs released by CRC cells increased. The team further showed that Hsp60 was also accumulated in CRC and pericancerous tissues (44). The level of Hsp60 on the membrane of blood-derived sEVs in postoperative patients with CRC was significantly lower than that in the same patients before removal of the tumors, and could be reduced to the level of healthy controls. This suggests that Hsp60^+^ sEVs may be a new marker of CRC.

#### Lung Cancer

Lung cancer is the most common type of cancer. The membrane of sEVs secreted from lung cancer tissue contained EGFR (95). EGFR^+^ sEVs can inhibit the function of tumor-specific CD8^+^ T cells through induced Tregs, thus accelerating the progression of lung cancer. Three membrane proteins, CD151, CD171 and TSPAN8, on the sEVs derived from lung cancer tissues can be used to distinguish different pathological types of lung cancer (96). In patients with unclear tissue subtypes of lung cancer, the AUC of detection of CD151 was 0.68, CD171, 0.61, and TSPAN8, 0.60. In patients with lung adenocarcinoma (AC), the AUC of CD171 was 0.63, TSPAN8 0.60, and CD151 0.67. In patients with squamous cell carcinoma of the lung (SCC) or small cell lung cancer (SCLC), CD151 was the only marker (AUC ≤ 0.69 in SCC and AUC ≤ 0.71 in SCLC).

37 protein markers were found in sEV of advanced non-small cell lung cancer (97). Univariate analysis showed that these proteins were significantly increased in patients with lung cancer. Multivariate analysis revealed that the model with 30 markers had an AUC value of 0.83. Recently, Li et al. (98) found that leucine-rich α-2-glycoprotein 1 (LRG1) was overexpressed on the membrane of urinary sEV and in cancer tissues of patients with non-small cell lung cancer (NSCLC). The LRG1 of sEVs may come from tumor tissues and may be a diagnostic marker for non-small cell lung cancer. Additionally, CD91, a serum-released sEV membrane protein, may be used as a detection index of lung adenocarcinoma (99). The AUC of CD91 and CEA were 0.724 and 0.794 respectively, and the AUC was 0.882 when they were combined. CD91 (54.5%) showed higher sensitivity than CEA (22.7%) in the detection of stage I and II of lung adenocarcinoma. However, their sensitivities for patients with III and IV are similar (CD91: 61.4%, CEA: 66.3%).

#### Melanoma

Melanoma is a highly invasive and metastatic skin cancer. It is worth noting that integrin expressed on melanoma-derived sEVs can be used to predict cancer cells metastasis (100). sEVs expressing integrin α2 and αv facilitate melanoma cell migration to liver and brain by promoting formation of premetastatic niche, while sEVs expressing integrin α4 and β1 gave priority to lymph node metastasis. Besides, PD-L1 was also expressed on the membrane of sEVs derived from metastatic melanoma (101). It can inhibit the activation of CD8^+^ T cells and promote the progression of melanoma, which may serve as a potential biomarker for melanoma.

#### Head and Neck Cancer

Theodoraki et al. (63) reported that the progression of patients with head and neck squamous cell carcinoma (HNSCC) was positively correlated with the number of PD-L1^+^ sEVs in plasma and the high expression of PD-L1 on sEVs. PD-L1 on these vesicles may inhibit the anti-tumor response by down-regulating the expression of CD69 on effector T cells. Ebnoether et al. (102) showed that the expression of plasma-released sEV membrane protein TGF-β was increased in patients with active head and neck cancer (HNC), and the expression in patients with III/IV stage was higher than that in I/II stage, which may serve as a progressive marker of HNC.

#### Summary of sEV Membrane Proteins as Biomarkers for Multiple Human Cancers

The aforementioned studies have indicated that sEV membrane proteins can be used as diagnostic and prognostic indicators for a variety of human cancers (summarized in **Table 3**). The combined characterization of multiple sEV membrane proteins for human cancers can improve the accuracy of detection (83, 85, 96), which further strengthens the potential of these proteins as biomarkers. In determining cancer progression, sEV membrane proteins often perform better compared to traditional markers (86, 87, 99). And when combined with traditional markers, the accuracy of cancer diagnosis can be improved (87, 93, 99). In addition, high expression of sEV membrane proteins in cancer patients may predict a shorter postoperative survival time (89). Meanwhile, the levels of some surface proteins of cancer patients were reduced to be comparable to those of healthy individuals after surgery (92, 94). Therefore, sEV membrane proteins have a promising application as cancer biomarkers. However, due to the differences in isolation methods and sample types, the obtained sEVs have great heterogeneity, leading to poor reproducibility of results, which limits their further applications.

**Table 3:** sEV membrane proteins as biomarkers for cancers.

Membrane protein	Source	Isolation	Purify	AUC	Diagnosis	Prognosis	Ref.
Prostate cancer							
Avβ3 integrin	Plasma, cell	UC	IG	–	√	√	(35, 77, 78)
Trop-2	Cell	UC	DUC	–	√	√	(79)
PSA	Cell, plasma	SC	SC	–	√	–	(80, 81)
PSMA	Plasma	IC	–	–	√	–	(82)
EpCAM+EGFR+Survivin+IGF-1R	Urine/ Plasma	IC	–	–	√	–	(83)
TM256	Urine	SRC	–	0.87	√	–	(84, 85)
TM256+LAMTOR1	Urine	SRC	–	0.94	√	–	(84, 85)
Pancreatic cancer							
GPC1	Serum	UC	SDG	1.0	√	–	(86)
EphA2	Serum	CRG	CRG	0.94,0.92	√	–	(87)
CD44v6	Cell	UC	SDG	–	–	√	(88)
PD-L1	Cell/ Serum	CRG	–	–	–	√	(89)
Colorectal cancer							
Wnt4	Cell	CRG	–	–	–	√	(91)
GPC1	Tissue, cell, plasma	CRG	SDG	–	√	√	(92)
Copine III	Tissue, plasma	UC	–	0.791	√	√	(93)
Hsp60	Tissue, cell, plasma	UC	–	–	√	√	(44, 94)
Lung cancer							
EGFR	Tissue	UC	–	–	–	√	(95)
CD151	Plasma	EV Array	–	–	√	–	(96)
CD171	Plasma	EV Array	–	–	√	–	(96)
TSPAN8	Plasma	EV Array	–	–	√	–	(96)
LRG1	Urine	IC	–	–	√	–	(98)
CD91	Serum	anti-CD9 MSIA tips	–	0.724	√	–	(99)
Melanoma							
PD-L1	Cell, plasma	UC	CRG	–	√	√	(101)
Head and neck cancer							
PD-L1	Plasma	mini-SEC	–	–	–	√	(63)

UC, ultracentrifugation; IC, immunocapture; SRC, serial centrifugation; mini-SEC, mini size-exclusion chromatography; Ref., references; DUC, differential ultracentrifugation; SC, successive centrifugations; SDG, Sucrose density gradients; CRG, Commercial reagents; IG, Iodixanol gradient; √, Applicable; -, Not applicable.

### Targeted Therapy

Recent studies have suggested that sEV membrane proteins can be used as drug targets for cancer treatment.

#### Receptor Proteins on sEV

PD-L1 is an important membrane protein on sEVs released by some tumor cells. PD-L1 on sEV membrane can promote tumor cell immune escape by inhibiting T cell activity (57). PD-L1 knockout TRAMP-C2 (a syngeneic model of prostate cancer) cells cannot form tumors in mice (103). Interestingly, PD-L1^+^ sEVs treatment promoted the growth of these knockout tumor cells in mice. Notably, the authors also found that sEVs produced by colon cancer cells promoted the growth of colon cancer cells with PD-L1 knockout, and the survival time of mice was prolonged when the sEVs secreted by the cells was removed. GW4869 (an inhibitor of sEVs secretion) were able to effectively slow down the growth of these tumor cells by inhibiting the secretion of sEVs in mouse breast cancer cells (62). When the release of PD-L1^+^ sEVs was specifically inhibited, it was found that the growth of breast cancer cells was significantly reduced. Therefore, targeting PD-L1 on these sEVs membranes may be a potential strategy for cancer treatment.

Several studies discovered that the membrane protein HSP70 on sEV may also be a potential therapeutic target for some tumors. Chalmin et al. (104) demonstrated that overexpressed HSP70 on the sEV membrane secreted by some cancer cells was able to bind to Toll-like receptor 2 to activate myeloid-derived suppressor cells, consequently, inhibiting immune cell response and promoting tumor progression. Gobbo et al. (105) found that blocking the binding of HSP70 and TLR2 with oligopeptide A8, which bound to HSP70 on sEVs, was able to inhibit the growth of cancer cells.

In addition, the membrane proteins CD44v6 and Tspan8 from the sEVs of pancreatic cancer initiation cells can render non-tumor initiation cells the ability to promote the occurrence and development of pancreatic cancer (106). After CD44v6-knockdown pancreatic cancer cells were transplanted into mice, anti-Tspan8 antibodies was able to block the transmission of Tspan8^+^ sEVs information in mice, thus inhibiting the growth and spread of these tumor cells. This may provide a new option for the treatment of pancreatic cancer.

#### Accessory Proteins

Lamp2b is a membrane protein on sEV that can bind to some cell-targeted peptides. Targeted therapy can be achieved by loading some therapeutic drugs into these vesicles. One report (107) showed that RVG, a neuron-specific peptide, bound to Lamp2b on engineered DC-produced sEVs. When siRNA-BACE1, a therapeutic target for Alzheimer’s disease, was loaded into these sEVs, the modified sEVs significantly reduced the mRNA and protein levels of BACE1 in mouse nerve cells. This may represent a potential way of gene therapy for Alzheimer’s disease. Recently, some researchers have combined iRGD (α v integrin specific targeting peptide) with Lamp2b on the membrane of sEVs derived from DCs, and then loaded Dox (doxorubicin) to form an iRGD-sEVs-Dox complex. The modified sEVs can target αv integrin positive human breast cancer cells, melanoma cells and liver cancer cells, and inhibit their growth (108). Similarly, Imatinib (a drug for the treatment of BCR-ABL positive chronic myeloid leukemia) can be loaded into Lamp2b^+^ sEVs. These sEVs produced by modified HEK293T cells combined with interleukin-3 (IL3) fragments were able to target IL3 receptor-rich chronic myeloid leukemia mother cells and inhibit their growth (109). Moreover, sEVs released from cardiomyocytes (CDCs) can stimulate angiogenesis, induce endogenous cardiomyocyte proliferation and regulate cardiomyocyte apoptosis (110, 111). Mentkowski et al. (112) ligated cardiomyocyte-specific peptides with sEV membrane protein Lamp2b derived from cardiomyocytes. These modified sEVs were able to enhance the cardiac tropism of CDCs-sEVs, thereby increasing cardiomyocyte uptake and reducing its apoptosis. In short, the membrane protein Lamp2b on sEV plays an auxiliary role in the targeted therapy of some diseases.

Tian et al. (113) have developed a potential method for the treatment of ischemic stroke using sEVs. Specifically, the sEV membrane proteins released from mouse bone marrow mesenchymal stem cells were indirectly linked to a short peptide (RGDyK) with high affinity for integrin αvβ3 receptor. The resulting sEVs loaded with curcumin were able to target the cerebral ischemia loss site of the mouse model of middle cerebral artery occlusion and reperfusion, and significantly inhibited the inflammatory response and apoptosis in the lesion area. Furthermore, modified sEVs can also be potentially used for treatment of Duchenne muscular dystrophy (114). A recombinant sEV was created by linking PMO (a targeted drug for the treatment of Duchenne muscular dystrophy) and M12 (a muscle-targeted peptide) to CD63 on the sEV membrane. These sEVs, when injected to into mice with Duchenne muscular dystrophy, were able to increase the expression of anti-dystrophin and improve muscle function.

In general, sEVs linked to cell-specific peptides are loaded with therapeutic drugs, which are a very promising strategy to achieve targeted treatment of diseases.

#### Targeted Proteins

Recent studies have shown that sEVs secreted from NK cells play a crucial role in killing tumor cells. The sEVs can specifically bind to Fas receptors on T-cell leukemia, erythroleukemia, lymphoma, and other cancer cells through the membrane protein mFasL on their surface, and induce the death of these cells (115). But the specific mechanism is not clear. Low concentration of FasL on the membrane of sEV can activate caspase-8, caspase-3 and PARP30 in melanoma cells. These three factors trigger the extracellular apoptotic pathway, thus inhibiting the growth of melanoma (116). Besides, It has been shown that CXCR4 expressing sEVs derived from NK cells are able to target neuroblastoma cells. These vesicles loaded with let-7a, an anti-tumor microRNA, can be delivered to neuroblastoma cells and inhibit their growth (117). In general, the membrane protein FasL or CXCR4 on sEVs produced by NK cells as ligands may achieve targeted therapy for some tumors.

In addition, sEV membrane protein HSP70 from healthy human plasma can improve cardiac function in rats with myocardial infarction (43). These sEVs bind to toll-like receptor 4 on the surface of cardiomyocyte membrane through HSP70, leading to activation of p38MAPK. The activated p38 in turn phosphorylates HSP27, which reduce the infarct size of the heart of rats with myocardial infarction.

#### Summarizing the Roles of sEV Membrane Proteins in Targeted Therapy

**Figure 2:**
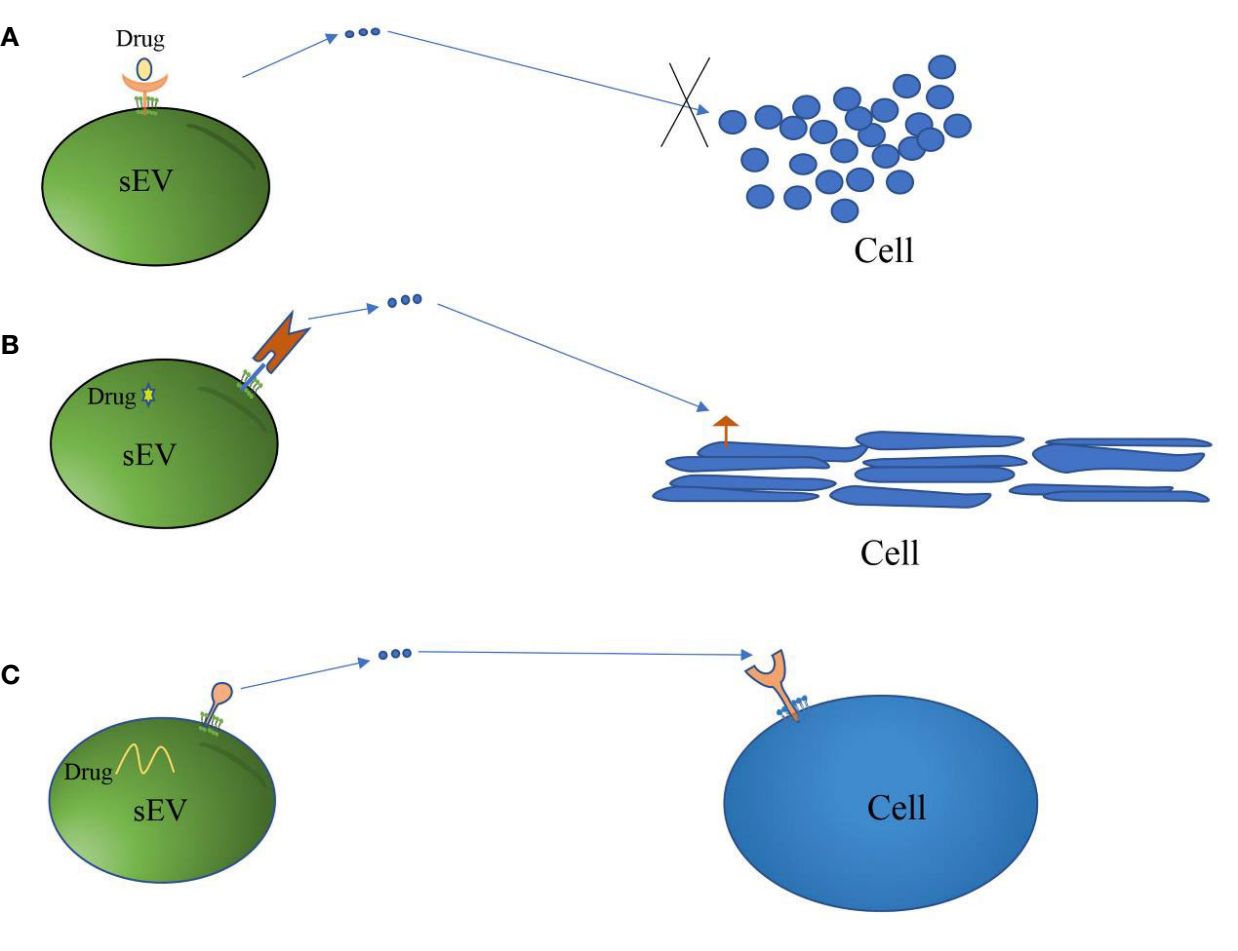
Mechanisms of sEV membrane proteins involved in targeted therapy. **(A)** Some sEV membrane proteins derived from tumor cells act as receptors of drugs. These drugs inhibit the growth or metastasis of cancer cells by blocking sEV-mediated information transmission. **(B)** sEV membrane proteins are linked to cell-specific proteins or peptides that target sEV carrying therapeutic drugs to specific cells, thereby achieving the purpose of targeted therapy. **(C)** sEV membrane proteins act as ligands to guide sEV carrying therapeutic drugs or having therapeutic effects to target specific cells, thus achieving the purpose of treatment.

There is a great prospect for sEV membrane proteins as mediators for targeted therapy of diseases (as shown in **Figure 2**). These proteins play a critical role in achieving drug interventions and gene therapy as well as in targeting of therapeutically effective sEVs, including mediating the attachment of drugs-loaded sEVs to cell-specific peptides. Accordingly, the treatment of cancer by sEV membrane proteins will likely be an innovative therapeutic strategy.

## Discussion

In recent years, the characteristics and functions of sEV membrane proteins have been continuously unveiled. Some Tetraspanin proteins (CD81, CD63, etc.) and peripheral membrane proteins (Alix, etc.) participate in the sorting of sEV contents. These proteins are often used as the marker proteins of sEVs. These membrane proteins play an important function under both physiological and pathological conditions. Four transmembrane proteins (CD9, CD37, etc.) have antigen presentation function; HSPs and Wnt proteins can promote cardiac angiogenesis and protect the heart; and peripheral membrane proteins, such as tenascin C, are associated with osteogenesis. Additionally, some sEV membrane proteins are closely related to development and progress of some cancers. Among them, PD-L1 mediated tumor immune escape and immunotherapy resistance; Integrins promote specific metastasis of some tumors; GPC1, a biomarker of pancreatic cancer and CRC, accelerates the progression of CRC. An increasing number of studies have proven that targeting the membrane proteins of sEV is an effective approach for the treatment of tumors, which may provide a new strategy for clinical treatment of cancer.

Previous studies have focused on the contents of vesicles (nucleic acids and proteins). These molecules, which are released into the receptor cells to act, need to be quantified after the vesicles are separated and fragmented, and then be used in subsequent studies or to reflect health status. However, this approach is highly susceptible to contamination by soluble and other impurity molecules in body fluids, which can affect the accuracy of results. In contrast, sEV membrane molecules, especially the extremely abundant membrane proteins, exert functions in the initiating cells as well as between donor and recipient cells. It is also possible to detect certain membrane proteins directly in body fluids which is same as circulating tumor cells, resulting in a rapid, accurate assay. The advantages of using these proteins as biomarkers are obvious, which include: (1) Non-invasive and specific; (2) Stable existence in body fluids with a long half-life; (3) Capture of sEV membrane proteins by immune capture method for diagnosing without the need of separating sEVs; (4) The changes of some proteins during disease progress can reflect the pathophysiological state of the body effectively. At the same time, the advantages of targeted therapy with sEV membrane proteins include: (1) easily crossing the biological barrier; (2) acting as a ligand to interact with the plasma membrane for information transmission; (3) highly stable, non-immunogenic and non-toxic. Therefore, the use of sEV membrane proteins as a potentially useful new tumor biomarkers and participate in the targeted treatment of disease is attractive. Moreover, surface proteins of sEV can also be used for sEVs capture and enrichment or as a normalization factor to provide quantitative information of vesicles.

However, it remains a challenge to obtain pure and homogenized sEVs for in-depth studies of membrane proteins, which limits clinical applications of sEV membrane proteins. What is exciting, is that a method has been developed for capturing sEVs directly from plasma, serum or urine using a variety of sEV membrane proteins. This method requires simple sample preparation without the need to separate vesicles (83). Therefore, the future research strategies of sEV membrane proteins may be divided into two types: (1) Isolation and purification of sEVs to further study sEV membrane proteins. This approach is limited by the difficulty in obtaining high-purity sEVs through the existing technology; (2) sEVs can be captured by immune capture method directly in body fluids, and used for analysis of membrane proteins. However, this method requires specific antibodies against membrane proteins, and the sensitivity of the antibodies used and possible reaction inhibitors will affect the accuracy of the results. Furthermore, there is obvious heterogeneity of sEVs in body fluid, so it is very important to trace their origins. The differential expression of some membrane proteins on these vesicles may be the basis of their origins. In the future, it is worth to determine whether sEVs in body fluids can be classified like blood cells based on their membrane proteins. If so, these sEVs can serve as a tool for diagnosis and treatment of diseases.

## Author Contributions

DFH and JC searched for literature and wrote the first draft of this article. DH and FX edited the manuscript. TY, ZL, and XW edited tables and figures. YX and JZ revised the manuscript. YJ, XZ, and TZ strictly reviewed the manuscript and polished the grammar. All authors contributed to the article and approved the submitted version.

## Funding

This work was supported by the National Natural Science Foundation of China [grant numbers 81702580], Key R&D Planning Project of Jiangxi Science and Technology Commission, China [No. 20203BBGL73126], and Creative Research Groups of Gannan Medical University, China [grant numbers TD201704].

## Conflict of Interest

The authors declare that the research was conducted in the absence of any commercial or financial relationships that could be construed as a potential conflict of interest.
